# Educational actions conducted during the pandemic with primary health care professionals: a scoping review

**DOI:** 10.1590/0034-7167-2023-0352

**Published:** 2024-08-30

**Authors:** Bruna Dias França, Kênia Lara Silva, Lilian Cristina Rezende, Francisco Carlos Felix Lana, Simone de Pinho Barbosa

**Affiliations:** I Universidade Federal de Minas Gerais. Belo Horizonte, Minas Gerais, Brazil; II Universidade Federal de Juiz de Fora. Governador Valadares, Minas Gerais, Brazil

**Keywords:** Education, Continuing, Health Personnel, Primary Health Care, COVID-19, Pandemics, Educación Continua, Personal de Salud, Atención Primaria de Salud, COVID-19, Pandemias

## Abstract

**Objectives:**

to map the educational actions conducted with primary health care professionals during the COVID-19 pandemic.

**Methods:**

a scoping review conducted in August 2023, which covered databases such as CINAHL, Medline, LILACS, IBECS, BDENF, and Web of Science. In total, 32 publications were analyzed through content analysis.

**Results:**

the primary beneficiaries of the educational actions included 69% physicians, 56% nurses, 25% pharmacists, 13% social workers and dentists, 9% psychologists, community health agents, and laboratory professionals, and 6% nursing technicians, nutritionists, and physical educators. The predominant educational interventions were training sessions (mentioned in 19 publications), followed by Continuing Health Education (10 publications) and Continuing Education (three publications).

**Final Considerations:**

the educational interventions demonstrated positive impacts on professional practice, particularly the Continuing Health Education actions, which were notable for stimulating critical problem-solving among professionals.

## RESUMEN

**Objetivos:**

mapear las acciones educativas realizadas con los profesionales de la salud de la Atención Primaria durante la pandemia de COVID-19.

**Métodos:**

revisión de alcance desarrollada en agosto de 2023, que abarcó las bases de datos CINAHL, Medline, LILACS, IBECS, BDENF y *Web of Science.* En total, se analizaron 32 publicaciones mediante análisis de contenido.

**Resultados:**

los principales beneficiarios de las acciones educativas incluyeron un 69% de médicos, un 56% de enfermeros, un 25% de farmacéuticos, un 13% de trabajadores sociales y dentistas, un 9% de psicólogos, agentes comunitarios de salud y profesionales de laboratorio, y un 6% de técnicos de enfermería, nutricionistas y educadores físicos. Las intervenciones educativas predominantes fueron los entrenamientos (mencionados en 19 publicaciones), seguidos por la Educación Permanente en Salud (10 publicaciones) y la Educación Continua (tres publicaciones).

**Consideraciones Finales:**

las intervenciones educativas demostraron impactos positivos en la práctica profesional, especialmente las acciones de Educación Permanente en Salud, que se destacaron por estimular la problematización crítica entre los profesionales.

## INTRODUCTION

Knowledge and expertise are continually updated in the healthcare field. To provide appropriate care, professionals need ongoing training that enhances skills for critical reasoning in their professional practice^(1)^. Professional competence is understood as a combination of knowledge, skills, and attitudes, developed to function in specific contexts and acquired through ongoing training^(2)^.

The World Health Organization (WHO) recognizes the necessity of investing in continuous training for professionals to improve the healthcare sector. It emphasizes the importance of process technology, which translates into knowledge and skills applied in the daily routines of professionals. To promote continuous development, it is essential to initiate educational actions that align with the demands of individuals and services, employ effective methods that meet the expectations of the subjects, and ensure access to suitable infrastructure, equipment, and tools^(3)^.

The global experience of the COVID-19 pandemic has vividly demonstrated the need for continuous professional training, especially during periods of uncertainty and high infection risk. This period led to increased hiring of healthcare personnel, including newly graduated and inexperienced professionals across various sectors, making work-oriented education even more vital to ensure the quality and safety of care^(3)^.

In Primary Health Care (PHC), which is crucial in communities and often serves as the first point of contact for healthcare services, continuous professional training is essential to meet the needs of territories, individuals, and communities^(4)^. PHC strives to provide universal, continuous, comprehensive, and equitable care, facilitating access and prioritizing health promotion, disease prevention, and the treatment of prevalent diseases^(5)^.

Amid the COVID-19 pandemic, with the urgent need for updates due to changing protocols and guidelines frequently redefined by the WHO, countries have invested in educational initiatives for PHC professionals^(6, 7)^. Resources such as simulation-based education^(8)^, health literacy^(9)^, counseling, monitoring, and training^(10)^, as well as the use of mobile electronic devices with internet access and experience sharing, have proven effective in educational efforts for these professionals^(10)^.

In Brazil, Permanent Health Education (PHE) actions are governed by the National Policy of Permanent Health Education (PNEPS), established in 2004. A core element of this policy is on-the-job learning, integrating learning and teaching into the daily operations of organizations. PHE serves as a tool to transform work practices, and its activities must pinpoint the learning needs of professionals based on and for their work^(11)^. From this perspective, digital platforms have been recognized as essential for the ongoing training of PHC workers, aiming to facilitate interprofessional communication in training processes during the pandemic^(10)^.

However, the process of on-the-job learning presents challenges that negatively impact the implementation of PHE, such as the underfunding of research expenses, work overload, long working hours, limited management involvement, low adherence of professionals to learning activities, a technicist culture in education, and bureaucracy^(12)^. Additionally, there are challenges related to the appreciation of formative initiatives that make teaching more flexible and responsive to the emerging needs of the workplace, as it is impractical to consider the development of workers’ skills apart from praxis. Thus, the teaching-learning process for professionals should aim to move beyond the logic of technical rationality towards a perspective that values individual experiences and the real challenges of territories and work scenarios^(13)^.

In light of the need for continuous training, which should be integrated into the daily routine of health services, and the challenges of its implementation, the guiding question of this study arises: “What evidence is available in the literature on educational actions carried out with PHC professionals for the development of professional competence during the COVID-19 pandemic?”. This study is justified by the need to map educational actions undertaken to develop professional competencies in PHC during the pandemic and to analyze whether these actions were effective in developing the necessary skills for professionals.

To assess the feasibility of conducting this review, a preliminary search was performed in the International Prospective Register of Systematic Reviews (PROSPERO) and the National Library of Medicine National Institutes of Health (PubMed). No review studies addressing this theme were identified, due to its recent context, which is rooted in the COVID-19 health crisis. The dissemination of lessons learned can contribute to better preparation of PHC professionals for future crises.

It is also emphasized that more studies are needed in the field of work-based education, due to the scarcity of literature on the topic, with the aim of filling this knowledge gap^(14)^ concerning educational actions in the workplace. It is hoped that the formative experiences provided by the COVID-19 pandemic will enhance understanding of the potentialities and weaknesses in work-based training and, from this, lead to improvements in the educational actions developed in this context. In the case of Brazil, the results of this study may provide new insights that assist in the operationalization and enhancement of PNEPS. Furthermore, the review aims to contribute to the sharing and dissemination of successful formative experiences in the health field.

## OBJECTIVES

To map the educational actions undertaken with primary healthcare professionals during the COVID-19 pandemic.

## METHODS

### Ethical Aspects

Given the nature of a scoping review, this study was not submitted for review to an ethics committee.

### Type of Study

This is a scoping review conducted in accordance with the Joanna Briggs Institute (JBI) Manual for Evidence Synthesis^(15)^ and the international PRISMA-ScR guide^(16)^. The review protocol was registered on the Open Science Framework (OSF) (https://doi.org/10.17605/OSF.IO/4AG7S)). Scoping reviews are utilized to map concepts or topics across various knowledge areas, aiming to identify and clarify existing gaps.

They are particularly useful for compiling evidence from diverse and heterogeneous sources^(15)^. According to the JBI manual (15), the review was executed in nine stages: (1) Development of the title, objective, and research question; (2) Definition of inclusion criteria aligned with the objective and research question; (3) Development of the research strategy; (4) Data collection; (5) Selection of identified studies; (6) Organization of selected studies; (7) Data analysis; (8) Presentation of results; (9) Summary of conclusions.

The PCC (Population, Concept, and Context) strategy was used to formulate the review question: P: Primary Health Care professionals; C: educational actions performed for the development of professional competence; C: context of the COVID-19 pandemic. The guiding question formulated was: “What evidence is available in the literature on educational actions carried out with PHC professionals for the development of professional competence during the COVID-19 pandemic?”.

### Data Collection and Organization

The search was conducted on August 28, 2023, across the following electronic databases: Medical Literature Analysis and Retrieval System Online (Medline), via PubMed; Spanish Bibliographic Index in Health Sciences (IBECS), Latin American and Caribbean Literature on Health Sciences (LILACS), Nursing Database (BDENF), via Virtual Health Library (BVS); Institute for Scientific Information (Web of Science), and Cumulative Index to Nursing and Allied Health Literature (CINAHL), accessed through the CAPES Periodicals Portal, using identification from the Federated Academic Community (CAFe). The search strategy was developed using descriptors and synonyms registered in the Health Sciences Descriptors (DeCS) and Medical Subject Headings (MeSH), including “Education, Continuing”, “Professional Training”, “Health Human Resource Training”, “Health Personnel”, “Primary Health Care”, “Community Health Services”; “COVID-19” (Chart 1).

**Chart 1:** Search Strategies in the Literature, Belo Horizonte, Minas Gerais, Brazil, 2023

Database	Library	Descriptors	Strategy
Medline*	National Library of Medicine National Institutes of Health (PubMed)	Education, Continuing / Professional Training / Health Human Resource Training / Health Personnel / Primary Health Care / Community Health Services / COVID-19	(“Education, continuing” [All Fields] OR “Professional Training” [All Fields] OR “Health Human Resource Training” [All Fields] OR “Health Personnel” [All Fields]) AND (“Primary Health Care” [All Fields] OR “Community Health Services” [All Fields]) AND “COVID-19” [All Fields]
IBECS**	Biblioteca Virtual de Saúde (BVS)	Education, Continuing / Professional Training / Health Human Resource Training / Health Personnel / Primary Health Care / Community Health Services / COVID-19	(“Education, Continuing” OR “*Educação continuada” OR “Educación Continua”* OR “Professional Training” OR “*Capacitação Profissional”* OR “*Capacitación Profesional”* OR “Health Human Resource Training” OR “*Capacitação de Recursos Humanos em Saúde”* OR “*Capacitación de Recursos Humanos en Salud”* OR “Health Personnel” OR “*Pessoal de Saúde”* OR “Personal de Salud”) AND (“Primary Health Care” OR “*Atenção Primária à Saúde”* OR “*Atención Primaria de Salud”* OR “Community Health Services” OR “*Serviços de Saúde Comunitária”* OR “*Servicios de Salud Comunitaria”*) AND (“COVID-19”)
LILACS***			
BDENF****			
Web of science	Web of science	Professional Training / Primary Health Care / COVID-19	((ALL=(Professional Training)) AND ALL=(Primary Health Care)) AND ALL=(COVID-19)
CINHAL*****	CINHAL*****		TX ( MH Professional Training OR Professional Training ) AND TX (MH Primary Health Care OR Primary Health Care) AND TX (MH COVID-19 OR COVID-19)

* Medical Literature Analysis and Retrieval System Online; **Índice Bibliográfico Español en Ciencias de la Salud; ***Literatura Latino-Americana e do Caribe em Ciências da Saúde; ****Base de Dados de Enfermagem; *****Cumulative Index to Nursing and Allied Health Literature.

### Search Strategy

A search strategy was employed for the LILACS, IBECS, and BDENF databases, given that these databases are integrated into a single library system, the BVS (Virtual Health Library). All strategies were developed with the assistance of a librarian. The following inclusion criteria were applied: all publications addressing the topic of educational actions for PHC professionals during the pandemic period, with no restrictions on publication period or language. Studies whose titles and abstracts did not meet the population, concept, and context requirements were initially excluded; the studies selected for full reading were evaluated against the same criteria, and those that did not meet them were excluded, as per the protocol designed for this review.

The first stage of the review involved searches in the databases using the strategies defined for each database. The results were exported to Microsoft Excel and later to EndNote, where duplicates were identified and removed. Titles and abstracts were then reviewed. In the second stage, the full texts of the articles selected in the first stage were read. Data collected from the full-text reading were organized using a tool with the items proposed by the JBI ^(15)^, which includes identification of the article, year and location of the study, context, participants, methodological characteristics, assessment of methodological rigor, and discussions about the thematic focus of this review.

Variables included about the thematic focus of this review were: type of educational action, formative need, topics discussed, tools used, and main results. Eligible studies were summarized by two researchers independently. Any discrepancies that arose between reviewers were resolved through discussion with a third reviewer. Information extracted from the publications was tabulated for the descriptive synthesis of the data.

### Data Analysis

The final sample of articles was subjected to content analysis. Initially, the educational actions described in the publications for PHC professionals were identified. After this initial identification, codes were created to characterize the teaching methodology used, a structuring element for the educational action, as it can provide favorable or unfavorable conditions for the development of competencies. Publications were then coded. Independent coding was carried out by two researchers. The described analysis process was conducted with the support of MaxQDA software, version 2022.

## RESULTS

**Figure 1:**
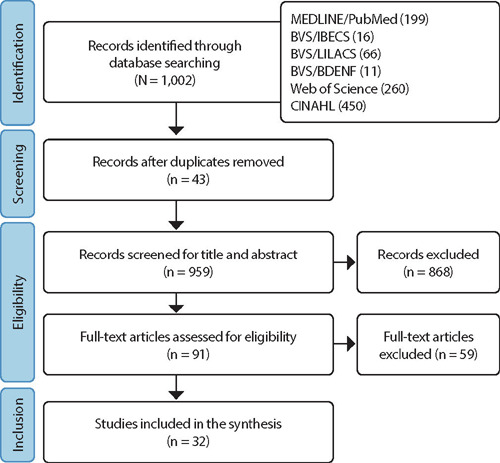
PRISMA Flowchart for Scoping Reviews (PRISMA-ScR) of the Literature Search Process

The database search identified 1,002 publications for screening, of which 43 were duplicates. Of the remaining 959 abstracts, 868 were excluded for not meeting the study’s objectives. Of the 91 full texts assessed for eligibility, a total of 59 studies were excluded because they did not present educational actions for the development of professional competencies in PHC. Overall, 32 studies were included as the final result of the investigation, comprising 29 articles in scientific journals, one dissertation, and two books found in the grey literature. The selection of articles was presented in the PRISMA-ScR flowchart^(16)^, Figure 1.

The 32 publications included in this review are presented in Chart 2, with the identification code, study reference, origin, type of study, level of evidence, and degree of recommendation according to the JBI Levels of Evidence for Effectiveness^(17)^ and population. The levels of evidence are determined by the type of study: 1 - experimental studies having the highest level of evidence, followed by 2 - quasi-experimental studies; 3 - analytical observational studies; 4 - descriptive and observational studies; and 5 - expert opinion studies and bench research, which have the lowest. The degree of recommendation, on the other hand, is related to the relevance and the method used to develop each type of study, identified with letters (a, b, c, d).

**Chart 2:** Characterization of the Publications (n=32), Belo Horizonte, Minas Gerais, Brazil, 2023

Study Reference	Origin	Type of Study	LE	Population
Santos et al. (2022)^(18)^	Brazil	Experience Report	4	Physician
Carvalho et al. (2022)^(19)^	Brazil	Experience Report	4	Community Health Worker
Schweickardt et al. (2022)^(20)^	Brazil	Experience Report	4	Physician, Nurse, and Community Health Worker
Silva et al. (2022)^(21)^	Brazil	Case Study	4D	Physician and Nurse
Cavalcanti et al. (2022)^(22)^	Brazil	Experience Report	4	Physician
Teixeira et al. (2022)^(23)^	Brazil	Experience Report	4	Nurse, Dentist, Nutritionist, Pharmacist, Psychologist, Physical Educator, and Social Worker
Santos et al. (2022)^(24)^	Brazil	Experience Report	4	Dentist
Iheanacho et al. (2021)^(25)^	Nigeria	Cross-sectional Study	4B	Physician, Nurse, Pharmacist, and Laboratory Professional
Odusanya et al. (2022)^(7)^	Nigeria	Quasi-experimental Study	2D	Physician, Nurse, Laboratory Professional, and Community Health Worker
Moura et al. (2021)^(26)^	Brazil	Exploratory Study	4	Nurse and Nursing Technician
Albahri et al. (2021)^(27)^	United Arab Emirates	Cross-sectional Study	4B	Nurse and Physician
Sumiya et al. (2021)^(28)^	Brazil	Cross-sectional Study	4B	Physiotherapist, Nurse, Nursing Technician, Dentist, Community Health Worker, Psychologist, Social Worker, Pharmacist, Speech Therapist, Physician, Occupational Therapist, and Physical Educator
Steeves-Reece et al. (2021)^(29)^	United States	Experience Report	4	Physician and Nurse
Jenkins et al. (2022)^(30)^	Ireland	Case Study	4C	Health Professionals and Social Worker
Phiri et al. (2022)^(31)^	Malawi	Mixed Methods Study	4	Health Professionals
Lau et al. (2021)^(32)^	Singapore	Cross-sectional Study	4B	Physician
Nasrallah et al. (2020)^(33)^	Qatar	Experience Report	4	Physician
Katzman et al. (2021)^(34)^	United States	Experience Report	4	Physician, Social Worker, Nurse, Community Health Worker, Pharmacist, Psychologist, and Nutritionist
Azimirad et al. (2023)^(35)^	Finland	Systematic Integrative Review	4A	Nurse
Downie et al. (2022)^(36)^	Tanzania	Feasibility Study	4	Nurse and Physician
Bidwell et al. (2022)^(37)^	New Zealand	Experience Report	4	Physician, Nurse, and Pharmacist
Harris et al. (2022)^(38)^	England	Experience Report	4	Physician, Nurse, Pharmacist, and Community Health Worker
Lau et al. (2021)^(39)^	Singapore	Cross-sectional Study	4B	Physician
Çevik et al. (2021)^(40)^	Turkey	Cross-sectional Study	4B	Physician
Adánez-Martínez et al. (2022)^(41)^	Spain	Cross-sectional Study	4B	Physician and Nurse
Alzahrani et al. (2022)^(42)^	Saudi Arabia	Cross-sectional Study	4B	Physician, Nurse, and Pharmacist
Bury et al. (2021)^(43)^	Ireland	Cross-sectional Study	4B	Physician, Nurse, and Other Primary Health Care Professionals
Klein et al. (2022)^(44)^	Argentina	Pilot Study	4	Physician, Nurse, and Community Health Worker
Peña-Galbán et al. (2022)^(45)^	Cuba	Experience Report	4	Health Professionals
Veras et al. (2021)^(46)^	Brazil	Experience Report	4	Health Professionals
Al-Khaldi et al. (2022)^(47)^	Saudi Arabia	Cross-sectional Study	4B	Physician, Nurse, Pharmacist, Laboratory Specialist, Dentist
Toledo-Ortiz et al. (2023)^(48)^	Mexico	Case Study	4C	Health Professionals

*LE - Level of Evidence; 2D - Quasi-experimental study (Pre-test and post-test); 4 - Descriptive and observational study; 4A - Systematic review; 4B - Descriptive observational study (Cross-sectional); 4D - Descriptive observational study (Case study).*

The type of study varied: 41% were experience repo rts^(18-20,22-24,29,33-34,37-38,45-46)^; 31% were cross-sectional studies^(25,27-28,32,39-43,47)^; 10% were case studies^(21,30,48)^; 3% were quasi-experimental^(7)^; 3% were systematic integrative reviews^(35)^; 3% were exploratory studies^(26)^; 3% were mixed methods studies^(31)^; 3% were feasibility studies^(36)^; and 3% were pilot studies^(44)^. Regarding the publication year, 3% of the studies were published in 2020^(33)^; 35% in 2021^(25-29,32,34,39-40,43,46)^; 56% in 2022^(7,18-24,30-31,36-38,41-42,44-45,47)^; and 6% in 2023^(35,48)^.

The populations targeted by the educational actions in PHC were: 69% physicians^(7,18,20-22,25,27-29,32-34,36-44,47)^; 56% nurses^(7,20-21,23,25-29,34-38,41-44,47)^; 25% pharmacists^(23,25,28,34,37-38,42,47)^; 13% social workers^(23,28,30,34)^, dentists^(23-24,28,47)^, 9% psychologists^(23,28,34)^, community workers^(7,34,44)^, Community Health Worker^(19-20,28)^ and laboratory professionals^(7,25,47)^; 6% nursing technicians^(26,28)^, nutritionists^(23,34)^ and physical educators^(23,28)^; 3% physiotherapists^(28)^, speech therapists^(28)^, and occupational therapists^(28)^.

**Figure 2:**
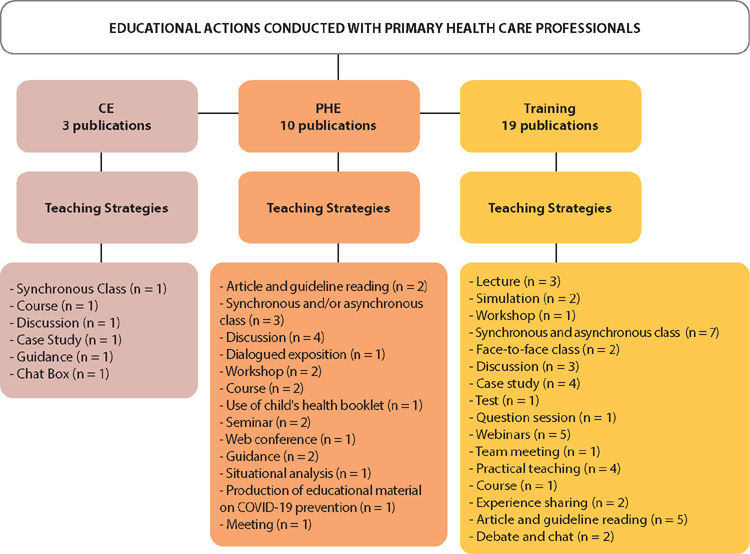
Map of Educational Actions Conducted with Primary Health Care Professionals and Their Teaching Strategies, Belo Horizonte, Minas Gerais, Brazil, 2023 PHC - Primary Health Care; CE- Continuing Education; PHE- Permanent Health Education; n = number of publications that cited the teaching strategy.

All studies were conducted considering the context of the COVID-19 pandemic. Regarding the concept, in the analysis process, it was possible to code the educational actions as illustrated in Figure 2, which presents the quantitative map of the sample on the educational actions carried out with PHC professionals and the teaching strategies used for these actions.

Training was conducted in 60% of the study sample^(7,25,27-33,36,38-45,47)^, followed by EPS with 31%^(18-24,26,46,48)^, and CE with 9%^(34-35,37)^.

The topics covered in the educational actions included: COVID-19^(7,18,20,22-24,28,31,33-36,40-41,43,45-48)^, Personal Protective Equipment (PPE)^(20,25-26,29,32-34,39,40,43,47)^, Infection Control^(25,27,32-33,39,42, 45,47)^, Organization of Work Processes During the Pandemic^(18,23,25)^, Non-Communicable Chronic Diseases (NCDs)^(35,38)^, Professional Ethics^(19,41)^, Epidemiology^(7,43)^, Mental Health^(19,23)^, Violence Against Women^(19,35)^, Senior Health^(19,29)^, Nutrition^(23,38)^, Telehealth Guidance^(29,33)^, Humanized Care^(20)^, Measles Surveillance Actions^(20)^, Neuromotor Assessment^(20)^, Child Development Surveillance^(21)^, Hand Hygiene^(23)^, Breathing Techniques^(23)^, Health Needs of a Community^(35)^, Remote Consulting^(36)^, and Introduction to PHC^(48)^.

**Figure 3:**
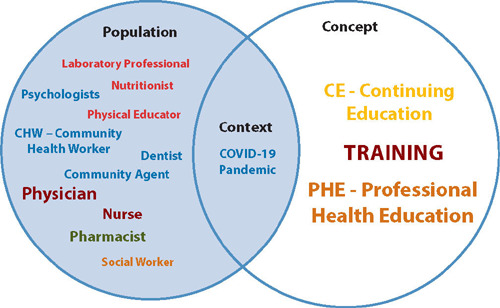
Representation of the Population, Concept, and Context, Belo Horizonte, Minas Gerais, Brazil, 2023 CE - Continuing Education; PHE - Permanent Health Education; CHW - Community Health Worker.

The integration of the work world with education became evident in the data, showcasing a diverse population of health professionals operating within the PHC setting, a unique context provided by the COVID-19 pandemic, and the implementation of the educational actions concept characterized by various educational activities, as demonstrated in Figure 3.

## DISCUSSION

The ongoing training of these professionals for a community-based approach is important for strengthening teamwork^(49)^. It is notable that physicians were the most prevalent category (69%) in the educational actions conducted, followed by nurses (56%). This result underscores the importance of involving other members of the primary healthcare team more extensively in educational activities to better qualify primary care. The implementation of educational actions for the development of professional competencies in primary healthcare during the context of COVID-19 has produced changes in the knowledge, skills, and attitudes of healthcare professionals^(7,25-28, 40,47)^. In uncertain times of a pandemic, the importance of these educational actions is recognized.

Training was conducted in 60% of the sample in this study, indicating a training model still centered on technicist formation^(49)^. The traditional training model, which refers to the transmission of strictly cognitive knowledge without necessarily linking theory and practice, uses more vertical and traditional educational methods that may be ineffective in promoting change^(50)^.

The most commonly used teaching strategy in training actions, as indicated in Figure 2, was synchronous and asynchronous classes^(7,28-30,36,41,45)^, followed by webinars^(25,28,30,33,40)^, reading articles and guidelines^(28,36,38,44,47)^, and practical teaching^(7,25,38,41)^. The most recurrent teaching strategies in training actions are configured in more traditional formats. Thus, it is assumed that the learning process in services presents challenges related to the format of educational action since training in the knowledge transmission format, following the traditional teaching model, may not represent a change in practice.

Studies^(25,27-28)^ also point to opinions of professionals who received training but did not feel fully prepared to act in the situation for which they were trained. Healthcare professionals should be educated to mobilize competencies and engage in critical thinking about their practice^(1)^. For this, it is necessary to consider educational actions with the potential to provoke this kind of thinking, seeking to develop in these professionals the knowledge, skills, and attitudes appropriate to operate in the work scenario.

However, it is emphasized that training was a way to improve the knowledge and skills of healthcare professionals worldwide, considering the territorial diversity in which it was carried out, and it helped professionals, especially in pandemic control^(27-33)^. Even though there was a need for other educational actions, it was noted that training improved the knowledge, skills, and attitudes of professionals, albeit moderately^(25,27-28)^.

In Brazil, PHE actions were utilized for training professionals in primary healthcare. This emphasis on PHE actions is understood to originate from its implementation as a National Policy since 2004^(11)^. PHE is considered a tool for problematizing the work process, beginning with an existing situation in pursuit of change, transformation, and overcoming it^(11)^. Regardless of the teaching strategy employed, PHE comprises resources that should contribute to and enable this problematization. It is notable that discussion^(18-19,22,23)^ was the most utilized teaching strategy in studies conducting this educational action, as depicted in Figure 2, as it allows reflection and problematization without being confined to traditional and hierarchical teaching strategies^(49-50)^.

The implementation of PNEPS in Brazil presents challenges in promoting problematizing debates about daily work, inadequate funding, lack of knowledge about the PNEPS baseline among managers and professionals, and replication of hegemonic educational practices of the traditional teaching model as PHE^(11)^. However, upon analyzing the educational actions in this review, the diversity of teaching strategies and their counter-hegemonic aspect compared to other presented actions are evident. Teaching strategies that contribute to problematization stand out, such as discussion^(18-19,22-23)^, workshop^(20,23)^, team meeting^(23)^, and situational analysis^(24)^. The openness to dialogue and problematization of work based on professionals’ prior knowledge are highlighted as differential elements compared to traditional teaching, which is based on information transmission. Dialogue is a resource that enables continuous exchange and construction of meanings through language^(50)^.

The strategic importance of PHE is recognized for strengthening and empowering workers in times of uncertainty by promoting continuous learning through reality problematization, thus contributing to the development of professional competencies^(18-24)^. Its territorial limitation is evident, presenting itself as a potent model for application in other countries.

In addition to training and PHE actions, CE initiatives were highlighted in the publications^(34-35,37)^. CE is understood as postgraduate training for professional updating, but it is not necessarily carried out to meet the needs of the work scenario like PHE. This action was carried out to update primary healthcare professionals on COVID-19, but it was initiatives existing before the pandemic, such as education programs for professionals, characteristic of this type of educational action. CE is not a commonly used educational action for developing competencies based on the needs of the work scenario. CE positively contributed in studies where it was mentioned for significant changes in professional practice in the primary healthcare scenario facing COVID-19. The teaching strategies used were synchronous class^(37)^, course^(37)^, discussion^(37)^, guidance^(35)^, case study^(34)^, and chat box^(34)^.

It’s important to highlight that in the three types of educational actions identified in this review, teaching strategies characteristic of a remote educational format were presented, including synchronous and asynchronous classes^(7,23,28-30,36-37,41,45-46,48)^, chat boxes^(29,33-34)^, webinars^(25,28,30,33,40)^, and web conferences^(22)^. The convenience and accessibility of online training were widely appreciated in the context of COVID-19 due to isolation and social distancing. However, the interpersonal connections possible in face-to-face format are considered irreplaceable^(35)^. It is worth noting that remote training during the COVID-19 pandemic enabled the implementation of educational actions where the facilitator/moderator and the learner could be in different geographical locations^(48)^, and provided tools that contribute to professionals’ learning in a quick and dialogical manner, such as the chat box. The remote modality allows for individual-paced learning and was necessary during the pandemic period due to social distancing^(25-35)^.

The various teaching strategies presented by the studies have the potential to develop competencies to deal with COVID-19, as they have a horizontal and dialogical format, allowing the professional to play a more active role in their learning process. Among these strategies, case studies, discussion, simulation, chat, and sharing experiences in discussions and practical teaching stand out. There is also evidence of the theme of COVID-19^(7,18,20,22-24,28,31,33-36,40-41,43,45-48)^ in Figure 3 as the focus in most publications, followed by related topics such as: ppe^(20,25-26,29,32-34,39-40,43,47)^, infection control^(25,27,32-33,39,42,45,47)^, organization of work processes during the pandemic^(18, 23,25)^, epidemiology^(7,43)^, guidance on telehealth^(29,33)^, hand hygiene^(23)^, and remote consulting^(36)^. This evidence was expected due to the context of the review.

Less frequently, themes such as NCDs^(35,38)^, mental health^(19,23)^, violence against women^(19,35)^, professional ethics^(19,41)^, nutrition^(23,38)^, elderly health^(19,29)^, humanized care^(20)^, measles surveillance actions^(20)^, neuromotor assessment^(20)^, child development surveillance^(21)^, breathing techniques^(23)^, community health needs^(35)^, and introduction to PHC^(48)^ were identified. Thus, it is important to highlight that PHC focuses on disease prevention and health promotion, with a focus on COVID-19, which may have left gaps in professionals’ knowledge about other topics in this scenario.

The evidence does not clearly indicate how competency development is evaluated or achieved. Therefore, the need for primary studies that seek to understand how these competencies are mobilized in PHC is emphasized. It is emphasized that crisis and post-crisis moments are considered potential for ruptures and advances in existing social practices.

### Study limitations

This study had limitations regarding the number of databases searched, which may have hindered access to other data.

### Contributions to the Health and Nursing Field

We believe that the evidence from this study can guide healthcare professionals and managers in maintaining, implementing, and improving the care offered to users through the promotion of contextualized and problematized learning actions by teams about their own work.

## FINAL CONSIDERATIONS

The educational actions conducted for the development of professional competencies mapped in this study included CE, PHE, and training. The findings prompt reflections on the importance of developing contextualized learning actions in the daily work routine. The potential of PHE for training and enhancing healthcare work is underscored, considering its learning methods that stimulate problematization about practice scenarios aiming at transforming both the professional and the practice itself. There is also a need for investment and strengthening of policies and programs, particularly in primary studies to understand the educational actions aimed at promoting continuous training of professionals for work in PHC. PHE was regarded as having the potential for problematization and the development of professional competencies for transforming practice at work. However, this concept is developed only within the Brazilian territory.
