# Supplementary material for: Educational actions conducted during the pandemic with primary health care professionals: a scoping review
Source: Rev Bras Enferm. 2024 Aug 30;77(Suppl 1):e20230352. doi: 10.1590/0034-7167-2023-0352 (PMC11368383; doi:10.1590/0034-7167-2023-0352)
Supplement: Translation

# Translation: Ações educativas realizadas na pandemia com profissionais de saúde da atenção primária: revisão de escopo

**DOI:** 10.1590/0034-7167-2023-0352pt

**Authors:** Bruna Dias França, Kênia Lara Silva, Lilian Cristina Rezende, Francisco Carlos Felix Lana, Simone de Pinho Barbosa

**Affiliations:** I Universidade Federal de Minas Gerais. Belo Horizonte, Minas Gerais, Brasil; II Universidade Federal de Juiz de Fora. Governador Valadares, Minas Gerais, Brasil

**Keywords:** Educação Continuada, Profissionais de Saúde, Atenção Primária, COVID-19, Pandemia

## Abstract

**Objetivos:**

mapear as ações educativas realizadas com os profissionais de saúde da Atenção Primária durante a pandemia da COVID-19.

**Métodos:**

revisão de escopo desenvolvida em agosto de 2023, abrangendo as bases *CINAHL, Medline,* LILACS, IBECS, BDENF e *Web of Science.* Ao todo, 32 publicações foram analisadas por meio de análise de conteúdo.

**Resultados:**

Os principais beneficiários das ações educativas incluíram 69% de médicos, 56% de enfermeiros, 25% de farmacêuticos, 13% de assistentes sociais e dentistas, 9% de psicólogos, agentes comunitários de saúde e profissionais de laboratório, e 6% de técnicos de enfermagem, nutricionistas e educadores físicos. As intervenções educacionais predominantes foram treinamentos (mencionados em 19 publicações), seguidas por Educação Permanente em Saúde (10 publicações) e Educação Continuada (três publicações).

**Considerações Finais:**

As intervenções educativas demonstraram impactos positivos na prática profissional, especialmente as ações de Educação Permanente em Saúde, que se destacaram por estimular a problematização crítica entre os profissionais.

## INTRODUÇÃO

Os conhecimentos e saberes são constantemente atualizados na área da saúde. Para oferecer cuidados de saúde apropriados, os profissionais necessitam de formação contínua, que mobilize competências para o raciocínio crítico em sua prática profissional^(1)^. A competência profissional é entendida como uma combinação de conhecimentos, habilidades e atitudes, desenvolvidas para atuar em um contexto específico e adquiridas em um processo de formação permanente^(2)^.

A Organização Mundial de Saúde (OMS) reconhece a necessidade de investimento e formação contínua dos profissionais para a melhoria do setor saúde. Destaca-se a importância da tecnologia de processos, que se traduz em saberes e habilidades aplicadas no cotidiano dos profissionais, ressaltando que para promover o desenvolvimento contínuo, deve-se estimular ações educativas em consonância com as demandas dos indivíduos e dos serviços, utilizar métodos eficazes e coerentes com as expectativas dos sujeitos, e garantir acesso a infraestruturas, equipamentos e ferramentas adequadas^(3)^.

A experiência global da pandemia da COVID-19 demonstrou concretamente a necessidade de formação profissional contínua, especialmente em períodos de incerteza e risco de infecção. Esse período demandou um aumento nas contratações de recursos humanos na saúde, incluindo profissionais recém-formados e inexperientes em diversos setores, tornando a educação voltada para o trabalho ainda mais essencial para garantir a qualidade e segurança da assistência^(3)^

Na Atenção Primária à Saúde (APS), que é de grande relevância nas comunidades e muitas vezes a porta de entrada para os cuidados em saúde, a formação profissional contínua é essencial para atender às necessidades dos territórios, indivíduos e coletividades^(4)^. A APS visa oferecer assistência universal, contínua, integral e equitativa, facilitando o acesso e priorizando a promoção da saúde, a prevenção de agravos e o tratamento de doenças prevalentes^(5)^.

No contexto da COVID-19, com a necessidade urgente de atualização devido à pandemia, que impôs novos protocolos e orientações redefinidos frequentemente pela OMS, os países investiram em ações educativas para os profissionais da APS^(6-7)^. Recursos como a educação baseada em simulação^(8)^, letramento em saúde^(9)^, aconselhamentos, monitoramento, treinamentos^(10)^, e o uso de dispositivos eletrônicos móveis com acesso à internet e compartilhamento de experiências foram considerados eficazes para as ações educativas dirigidas a esses profissionais^(10)^.

No Brasil, as ações de Educação Permanente em Saúde (EPS) são regulamentadas pela Política Nacional de Educação Permanente em Saúde (PNEPS), instituída em 2004, tendo como elemento essencial a aprendizagem no trabalho, na qual o aprender e o ensinar se incorporam ao cotidiano das organizações. A EPS é uma ferramenta de transformação das práticas no trabalho, e suas ações devem identificar as necessidades de aprendizagem dos profissionais a partir do trabalho e para o trabalho^(11)^. Nessa perspectiva, foram apontadas como fundamentais para a formação contínua dos trabalhadores da APS as plataformas digitais, buscando favorecer a comunicação interprofissional nos processos formativos em tempos de pandemia^(10)^.

Todavia, o processo de aprendizagem no trabalho apresenta desafios que contribuem negativamente para a implantação da EPS, como o subfinanciamento dos gastos com pesquisa, a sobrecarga de trabalho, jornadas de trabalho extensas, pouco envolvimento de gestores, baixa adesão dos profissionais às atividades de aprendizagem, a cultura tecnicista da educação e a burocracia^(12)^. Apontam-se ainda desafios relacionados à valorização de iniciativas formativas que dinamizem e flexibilizem o ensino a partir das necessidades emergentes do trabalho, uma vez que não há como pensar o desenvolvimento de competências dos trabalhadores de maneira desarticulada da práxis. Assim, o processo de ensino-aprendizagem dos profissionais deve buscar superar a lógica da racionalidade técnica por uma perspectiva que permita e valorize as experiências dos indivíduos e as reais dificuldades dos territórios e cenários de trabalho^(13)^.

Diante da necessidade de formação contínua, que deve ser motivada no cotidiano dos serviços de saúde, e dos desafios de sua implantação, surge a questão norteadora deste estudo: “Quais são as evidências disponíveis na literatura sobre as ações educativas realizadas com os profissionais de saúde da APS para o desenvolvimento de competência profissional durante a pandemia da COVID-19?”. O presente estudo se justifica pela necessidade de mapear as ações educativas realizadas para desenvolver as competências profissionais na APS durante a pandemia e analisar se essas foram capazes de desenvolver as competências necessárias para os profissionais.

Para avaliar a viabilidade da execução desta revisão, realizou-se uma busca preliminar no *International Prospective Register of Systematic Reviews* (PROSPERO) e na *National Library of Medicine National Institutes of Health* (PubMed). Não foram identificados estudos de revisão que abordassem esta temática, devido ao seu contexto recente, fundamentado na crise sanitária da COVID-19. A divulgação das lições aprendidas pode contribuir para que, no futuro, os profissionais da APS estejam melhor preparados para enfrentar outras crises.

Ressalta-se também a necessidade de mais estudos no campo da educação para o trabalho, devido à escassez de literatura sobre o tema, visando preencher essa lacuna de conhecimento^(14)^ sobre as ações educativas no ambiente de trabalho. Espera-se que as experiências formativas proporcionadas pela pandemia da COVID-19 contribuam para o entendimento das potencialidades e fragilidades na formação para o trabalho e, a partir disso, oportunizem melhorias nas ações educativas desenvolvidas nesse contexto. No caso do Brasil, os resultados deste estudo podem fornecer novas informações que auxiliem na operacionalização e aprimoramento da PNEPS. Além disso, a revisão visa contribuir para o compartilhamento e divulgação de experiências formativas exitosas no campo da saúde.

## OBJETIVOS

Mapear as ações educativas realizadas com os profissionais de saúde da Atenção Primária durante a pandemia da COVID-19.

## MÉTODOS

### Aspectos éticos

Dado o caráter de uma revisão de escopo, este estudo não foi submetido a um comitê de ética.

### Tipo de estudo

Este é um estudo de revisão de escopo, conduzido com base nos princípios do *Joanna Briggs Institute (JBI), Manual for Evidence Synthesis* ^(15)^ e pelo guia internacional PRISMA-ScR^(16)^. O protocolo de revisão foi registrado no Open Science Framework (OSF) (https://doi.org/10.17605/OSRIO/4AG7S). As revisões de escopo são empregadas para mapear conceitos ou tópicos em diversas áreas de conhecimento, visando identificar e esclarecer lacunas existentes. Elas são particularmente úteis para compilar evidências de fontes diversas e heterogêneas^(15)^.

Conforme o manual da JBI^(15)^, a revisão foi executada em nove etapas: (1) Elaboração do título, objetivo e questão de pesquisa; (2) Definição dos critérios de inclusão alinhados ao objetivo e à pergunta de pesquisa; (3) Desenvolvimento da estratégia de pesquisa; (4) Coleta de dados; (5) Seleção dos estudos identificados; (6) Organização dos estudos selecionados; (7) Análise dos dados; (8) Apresentação dos resultados; (9) Resumo das conclusões.

Utilizou-se a estratégia PCC (*Population, Concept, and Context*) para a formulação da questão de revisão, sendo: P: Profissionais de saúde da APS; C: ações educativas realizadas para o desenvolvimento de competência profissional; C: contexto da pandemia da COVID-19. A pergunta norteadora formulada foi: “Quais são as evidências disponíveis na literatura sobre as ações educativas realizadas com os profissionais de saúde da APS para o desenvolvimento de competência profissional durante a pandemia da COVID-19?”.

### Coleta e organização dos dados

A pesquisa foi realizada em 28 de agosto de 2023 nas seguintes bases de dados eletrônicas: *Medical Literature Analysis and Retrieval System Online (Medline), via PubMed; Índice Bibliográfico Español en Ciencias de la Salud (IBECS),* Literatura Latino-Americana e do Caribe em Ciências da Saúde (LILACS), Base de Dados de Enfermagem (BDENF), via Biblioteca Virtual de Saúde (BVS); *Institute for Scientific Information (Web of Science) e Cumulative Index to Nursing and Allied Health Literature (CINAHL),* acessadas através do Portal de Periódicos da CAPES, com uso da identificação da Comunidade Acadêmica Federada (CAFe). A estratégia de busca foi elaborada utilizando descritores e sinônimos registrados no Descritor em Ciências da Saúde (DeCS) e no *Medical Subject Headings (MeSH), incluindo: “Education, Continuing”; “Professional Training”; “Health Human Resource Training”; “Health Personnel”; “Primary Health Care”; “Community Health Services”; “COVID-19”* (Quadro 1).

**Quadro 1:** Estratégias de busca na literatura, Belo Horizonte, Minas Gerais, Brasil, 2023

Base de dados	Biblioteca	Descritores	Estratégia
Medline*	*National Library of Medicine National Institutes of Health* (PubMed)	*Education, Continuing/Professional Training/Health Human Resource Training/Health Personnel/Primary Health Care / Community Health Services/COVID-19*	*(“Education, continuing” [All Fields] OR “Professional Training” [All Fields] OR “Health Human Resource Training” [All Fields] OR “Health Personnel” [All Fields]) AND (“Primary Health Care” [All Fields] OR “Community Health Services” [All Fields]) AND “COVID-19” [All Fields]*
IBECS**	Biblioteca Virtual de Saúde (BVS)	*Education, Continuing/Professional Training / Health Human Resource Training / Health Personnel / Primary Health Care / Community Health Services/COVID-19*	*(“Education, Continuing” OR “*Educação continuada” *OR “Educación Continua” OR “Professional Training” OR “*Capacitação Profissional” *OR “Capacitación Profesional” OR “Health Human Resource Training” OR “*Capacitação de Recursos Humanos em Saúde” *OR “Capacitación de Recursos Humanos en Salud” OR “Health Personnel” OR “*Pessoal de Saúde” *OR “Personal de Salud”*) *AND* (“*Primary Health Care” OR “*Atenção Primária à Saúde” *OR “Atención Primaria de Salud” OR “Community Health Services” OR “*Serviços de Saúde Comunitária” *OR “Servicios de Salud Comunitaria”*) *AND* (“COVID-19”)
LILACS***			
BDENF****			
*Web of science*	*Web of science*	*Professional Training / Primary Health Care/COVID-19*	*((ALL=(Professional Training)) AND ALL=(Primary Health Care)) AND ALL=(COVID-19)*
CINHAL*****	CINHAL*****		*TX (MH Professional Training OR Professional Training) AND TX (MH Primary Health Care OR Primary Health Care) AND TX (MH COVID-19 OR COVID-19)*

* Medical Literature Analysis and Retrieval System Online; **Índice Bibliográfico Español en Ciencias de la Salud; ***Literatura Latino-Americana e do Caribe em Ciências da Saúde; ****Base de Dados de Enfermagem; *****Cumulative Index to Nursing and Allied Health Literature.

Foi utilizada uma estratégia de busca para as bases LILACS, *IBECS* e BDENF, considerando que essas bases estão integradas em uma única biblioteca, a BVS. Todas as estratégias foram construídas com o auxílio de uma bibliotecária. Foram aplicados os seguintes critérios de inclusão: todas as publicações que abordassem o tema de ações educativas para profissionais da APS durante o período de pandemia, sem limitação de período de publicação ou idioma. Foram inicialmente excluídos estudos cujos títulos e resumos não atendiam aos requisitos de população, conceito e contexto; os estudos selecionados para leitura na íntegra avaliaram os mesmos requisitos, e aqueles que não os atendiam foram excluídos, conforme o protocolo elaborado para esta revisão.

A primeira etapa da revisão envolveu buscas nas bases de dados por meio da aplicação das estratégias definidas para cada base. Os resultados foram exportados para os *softwares Microsoft Excel* e posteriormente para o *EndNote,* nos quais as duplicatas foram identificadas e excluídas. Em seguida, procedeu-se à leitura de títulos e resumos. Na segunda etapa, realizou-se a leitura na íntegra dos artigos selecionados na primeira etapa. Os dados coletados na leitura na íntegra foram organizados a partir de um instrumento com os itens propostos pelo JBI^(15)^, que contempla a identificação do artigo, ano e local do estudo, contexto, participantes, características metodológicas, avaliação do rigor metodológico, apontamentos e discussões acerca do foco temático desta revisão.

As variáveis incluídas acerca do foco temático desta revisão foram: tipo de ação educativa, necessidade formativa, temas discutidos, ferramentas utilizadas e principais resultados. Os estudos elegíveis foram sumarizados por dois pesquisadores de forma independente. Quaisquer divergências surgidas entre os revisores foram resolvidas por meio de discussão com um terceiro revisor. As informações extraídas das publicações foram tabuladas para a síntese descritiva dos dados.

### Análise dos dados

A amostra final de artigos foi submetida à análise de conteúdo. Inicialmente, foram identificadas as ações educativas descritas nas publicações para os profissionais da APS. Após essa identificação inicial, foram criados códigos para caracterizar a metodologia de ensino utilizada, um elemento estruturante para a ação educativa, já que pode proporcionar condições favoráveis ou não para o desenvolvimento de competências. Em seguida, realizou-se a codificação das publicações. A codificação independente foi realizada por dois pesquisadores. O processo de análise descrito foi realizado com o suporte do *software* MaxQDA versão 2022.

## RESULTADOS

A busca nos bancos de dados identificou ,002 publicações para triagem, das quais 43 eram duplicatas. Dos 959 resumos restantes, 868 foram excluídos por não atenderem ao objetivo do estudo. Dos 9, textos completos avaliados para elegibilidade, um total de 59 estudos foram excluídos por não apresentarem ações educativas para o desenvolvimento de competências profissionais na APS. No geral, 32 estudos foram incluídos como resultado final da investigação, sendo 29 artigos em periódicos científicos, uma dissertação e dois livros encontrados na literatura cinzenta. A seleção dos artigos foi apresentada no fluxograma PRISMA-ScR^(16)^, Figura 1.

**Figura 1:** Fluxograma PRISMA para revisões de escopo (PRISMA-ScR) do processo de busca na literatura

As 32 publicações incluídas nesta revisão são apresentadas no Quadro 2, com identificação do código, referência do estudo, origem, tipo de estudo, nível de evidência e grau de recomendação segundo o *JBI Levels of Evidence for Effectiveness* ^(17)^ e população. Os níveis de evidência são determinados pelo tipo de estudo, sendo: , - estudos experimentais, que possuem o mais alto nível de evidência, seguidos pelos 2 - quase-experimentais; 3 - estudos analíticos observacionais; 4 - estudos descritivos e observacionais; e 5 - estudos de opinião de especialista e pesquisas de bancada, que possuem o nível mais baixo. O grau de recomendação, por outro lado, está relacionado à relevância e ao método utilizado para desenvolver cada tipo de estudo, sendo identificados com letras (a, b, c, d).

**Quadro 2:** Caracterização das publicações (n=32), Belo Horizonte, Minas Gerais, Brasil, 2023

Referência do Estudo	Origem	Tipo de Estudo	NE	População
Santos et al. (2022)^(18)^	Brasil	Relato de experiência	4	Médico
Carvalho et al. (2022)^(19)^	Brasil	Relato de experiência	4	ACS
Schweickardt et al. (2022)^(20)^	Brasil	Relato de experiência	4	Médico, enfermeiro e ACS
Silva et al. (2022)^(21)^	Brasil	Estudo de caso	4D	Médico e enfermeiro
Cavalcanti et al. (2022)^(22)^	Brasil	Relato de experiência	4	Médico
Teixeira et al. (2022)^(23)^	Brasil	Relato de experiência	4	Enfermeiro, dentista, nutricionista, farmacêutico, psicólogo, Educador físico e assistente social
Santos et al. (2022)^(24)^	Brasil	Relato de experiência	4	Dentista
Iheanacho et al. (2021)^(25)^	Nigéria	Estudo transversal	4B	Médico, enfermeiro, farmacêutico e profissional de laboratório
Odusanya et al. (2022)^(7)^	Nigéria	Estudo quase experimental	2D	Médico, enfermeiro, profissional de laboratório e trabalhador comunitário de saúde
Moura et al. (2021)^(26)^	Brasil	Estudo exploratório	4	Enfermeiro e técnico de enfermagem
Albahri et al. (2021)^(27)^	Emirados Árabes	Estudo transversal	4B	Enfermeiro e Médico
Sumiya et al. (2021)^(28)^	Brasil	Estudo transversal	4B	Fisioterapeuta, enfermeiro, técnico de enfermagem, dentista, ACS, psicólogo, assistente social, farmacêutico, fonoaudiólogo, médico, TO e Educador físico
Steeves-Reece et al. (2021)^(29)^	Estados Unidos	Relato de experiência	4	Médico e enfermeiro
Jenkins et al. (2022)^(30)^	Irlanda	Estudo de caso	4C	Profissionais de saúde e assistente social
Phiri et al. (2022)^(31)^	Malawi	Estudo de métodos mistos	4	Profissionais de saúde
Lau et al. (2021)^(32)^	Singapura	Estudo transversal	4B	Médico
Nasrallah et al. (2020)^(33)^	Catar	Relato de experiência	4	Médico
Katzman et al. (2021)^(34)^	Estados Unidos	Relato de experiência	4	Médico, assistente social, enfermeiro, agente comunitário, farmacêutico, psicólogo e nutricionista
Azimirad et al. (2023)^(35)^	Finlândia	Revisão Integrativa Sistemática	4A	Enfermeiro
Downie et al. (2022)^(36)^	Tanzânia	Estudo de viabilidade	4	Enfermeiro e médico
Bidwell et al. (2022)^(37)^	Nova Zelândia	Relato de experiência	4	Médico, enfermeiro e farmacêutico
Harris et al. (2022)^(38)^	Inglaterra	Relato de experiência	4	Médico, enfermeiro, farmacêutico e agente comunitário
Lau et al. (2021)^(39)^	Singapura	Estudo transversal	4B	Médico
Çevik et al. (2021)^(40)^	Turquia	Estudo transversal	4B	Médico
Adánez-Martínez et al. (2022)^(41)^	Espanha	Estudo transversal	4B	Médico e enfermeiro
Alzahrani et al. (2022)^(42)^	Arábia Saudita	Estudo transversal	4B	Médico, enfermeiro e farmacêutico
Bury et al. (2021)^(43)^	Irlanda	Estudo transversal	4B	Médico, enfermeiro e outros profissionais da APS
Klein et al. (2022)^(44)^	Argentina	Estudo piloto	4	Médico, enfermeiro e agente comunitário
Peña-Galbán et al. (2022)^(45)^	Cuba	Relato de experiência	4	Profissionais de saúde
Veras et al. (2021)^(46)^	Brasil	Relato de experiência	4	Profissionais de saúde
Al-Khaldi et al. (2022)^(47)^	Arábia Saudita	Estudo transversal	4B	Médico, enfermeiro, farmacêutico, Especialista em laboratório, Dentista.
Toledo-Ortiz et al. (2023)^(48)^	México	Estudo de caso	4C	Profissionais de saúde

*NE - Nível de Evidência; ACS - Agente Comunitário de Saúde; TO - Terapeuta Ocupacional; 2D - Estudo quase-experimental (Pré-teste e pós-teste); 4 - Estudo descritivo e observacional; 4A - Revisão sistemática; 4B - Estudo descritivo observacional (Transversal); 4D - Estudo descritivo observacional (Estudo de caso).*

O tipo de estudo variou em relato de experiência (41%)^(18-20,22-24,29,33-34,37-38,45-46)^; transversal (31%)^(25,27-28,32,39-43,47)^; estudo de caso (10%)^(21,30,48)^; quase experimental (3%)^(7)^; Revisão integrativa sistemática (3%)^(35)^; estudo exploratório (3%)^(26)^; estudo de métodos mistos (3%)^(31)^; estudo de viabilidade (3%)^(36)^; estudo piloto (3%) ^(44)^. Quanto ao ano de publicação, 3% dos estudos foram publicados em 2020^(33)^; 35% foram publicados em 2021^(25-29,32,34,39-40,43,46)^; 56% foram publicados em 2 0 22^(7,18-24,30-31,36-38,41-42,44-45,47)^; 6% foram publicados em 2023^(35,48)^.

Os textos analisados apresentaram como população das ações educativas na APS: 69% médicos^(7,18,20-22,25,27-29,32-34,36-44,47)^; 56% enfermeiros^(7,20-21,23,25-29,34-38,41-44,47)^; 25% farmacêuticos^(23,25,28,34, 37-38,42,47)^, 13% assistente social^(23,28,30,34)^, dentistas^(23-24,28,47)^, 9% psicólogos^(23,28,34)^, agentes comunitários^(7,34,44)^, ACS^(19-20,28)^ e profissional de laboratório^(7,25,47)^; 6% Técnico de enfermagem^(26,28)^, nutricionista^(23,34)^ e educador físico^(23,28)^; 3% Fisioterapeuta^(28)^, Fonoaudiólogo^(28)^ e TO^(28)^.

Todos os estudos foram conduzidos considerando o contexto da pandemia da COVID-19. Em relação ao conceito, no processo de análise, foi possível codificar as ações educativas conforme ilustrado na Figura 2, que apresenta o mapa quantitativo da amostra sobre as ações educativas realizadas com os profissionais da APS e as estratégias de ensino utilizadas para essas ações.

**Figura 2:** Mapa das ações educativas realizadas com os profissionais da Atenção Primária à Saúde e suas estratégias de ensino, Belo Horizonte, Minas Gerais, Brasil, 2023 APS - Atenção Primária à Saúde; EC - Educação Continuada; EPS - Educação Permanente em Saúde; n = número de publicações que citou a estratégia de ensino.

O treinamento foi realizado em 60% da amostra deste estudo^(7,25,27-33,36,38-45,47)^ seguido pela EPS com 31%^(18-24,26,46,48)^ e pela Educação continuada (EC) com 9%^(34-35,37)^.

Os temas abordados nas ações educativas foram: COVID-19^(7,18,20,22-24,28,31,33-35,36,40-41,43,45-48)^, Equipamento de Proteção Individual (EPI)^(20,25-26,29,32-34,39-40,43,47)^, Controle de infecção^(25,27,32-33,39,42,45,47)^, Organização do processo de trabalho frente à pandemia^(18,23,25)^, Doenças Crônicas não Transmissíveis (DCNT’s)^(35,38)^, Ética profissional^(19,41)^, Epidemiologia^(7,43)^, Saúde mental^(19,23)^, Violência contra a mulher^(19,35)^, Saúde do idoso^(19,29)^, Alimentação^(23,38)^, Orientação sobre telessaúde^(29,33)^, Acolhimento humanizado^(20)^, Ações de vigilância em sarampo^(20)^, Avaliação neuromotora^(20)^, Vigilância do Desenvolvimento Infantil^(21)^, Higienização das mãos^(23)^, Técnicas de respiração^(23)^, Necessidades de saúde de uma comunidade^(35)^, Consultoria remota^(36)^ e Introdução a APS^(48)^.

Ao analisar a integração do mundo do trabalho com a educação ficou evidente nos dados uma população variada de profissionais da saúde que atuam no cenário da APS, um contexto singular proporcionado pela pandemia da COVID-19 e a implementação do conceito de ações educativas caracterizado por variadas ações educativas, conforme demonstrado na Figura 3.

**Figura 3:** Representação do Population, Concept, and Context, Belo Horizonte, Minas Gerais, Brasil, 2023 EC - Educação Continuada; EPS - Educação Permanente em Saúde; ACS - Agente Comunitário de Saúde.

## DISCUSSÃO

A formação contínua desses profissionais para uma abordagem comunitária é importante para o fortalecimento do trabalho em equipe^(49)^. Nota-se que o médico foi a categoria mais prevalente (69%) nas ações educativas realizadas, seguido pelos enfermeiros (56%). Este resultado reforça a importância de envolver mais os demais membros da equipe de saúde da APS nas ações educativas a fim de melhor qualificar a atenção primária. A realização de ações educativas para o desenvolvimento de competências profissionais na APS no contexto da COVID-19 produziu mudanças nos conhecimentos, habilidades e atitudes dos profissionais de saúde^(7,25-28,40,47)^. Em momentos incertos de pandemia, reconhece-se a importância dessas ações educativas.

O treinamento foi realizado em 60% da amostra deste estudo, o que demonstra um modelo formativo ainda centrado na formação tecnicista^(49)^. O modelo de treinamento tradicional, que se refere à transmissão de conhecimento de natureza estritamente cognitiva, sem necessariamente uma articulação entre teoria e prática, utiliza métodos educacionais mais verticais e tradicionais, que podem ser pouco eficazes em promover mudanças^(50)^.

A estratégia de ensino mais utilizada nas ações de treinamento, conforme indicado na Figura 2, foi a aula síncrona e assíncrona^(7,28-30,36,41,45)^, seguida por webinars^(25,28-30,33,40)^, leitura de artigos e diretrizes^(28,36,38,44,47)^ e ensino prático^(7,25,38,41)^. As estratégias de ensino mais recorrentes em ações de treinamento configuram-se em formatos mais tradicionais. Assim, pressupõe-se que o processo de aprendizagem nos serviços apresenta desafios relacionados ao formato de ação educativa, uma vez que o treinamento no formato de transmissão de conhecimento, seguindo o modelo tradicional de ensino, pode não representar uma mudança na prática.

Os estudos^(25,27-28)^ apontam ainda opiniões de profissionais que receberam treinamento mas não se sentiram totalmente preparados para atuar frente à situação para a qual foram treinados. Os profissionais de saúde devem ser educados para mobilizar competências e engajar-se no raciocínio crítico de sua prática^(1)^. Para isso, é necessário pensar em ações educativas com potencial para provocar esse raciocínio, buscando desenvolver nesses profissionais conhecimentos, habilidades e atitudes adequadas para atuar no cenário de trabalho.

Ressalta-se, contudo, que o treinamento foi uma maneira de melhorar o conhecimento e as habilidades dos profissionais de saúde em todo o mundo, considerando a diversidade territorial em que foi realizado, e ajudou os profissionais, especialmente no controle da pandemia^(27-33)^. Mesmo que houvesse a necessidade de outras ações educacionais, foi apontado que o treinamento melhorou os conhecimentos, habilidades e atitudes dos profissionais, ainda que moderadamente^(25,27-28)^.

No Brasil, as ações de EPS foram utilizadas para a formação dos profissionais na APS. Entende-se que esse destaque para as ações de EPS deve-se à sua implementação como uma Política Nacional desde 2004^(11)^. A EPS é considerada um dispositivo para problematização sobre o processo de trabalho, partindo de uma situação existente em busca de mudá-la, transformá-la e superá-la^(11)^. Independentemente da estratégia de ensino utilizada, a EPS configura-se em recursos que devem contribuir e proporcionar essa problematização. Destaca-se que a discussão^(18-19,22-23)^ foi a estratégia de ensino mais utilizada nos estudos que realizaram essa ação educativa, conforme demonstrado na Figura 2, uma vez que permite refletir e problematizar sem se limitar a estratégias de ensino tradicionais e hierarquizadas^(49-50)^.

A implementação da PNEPS no Brasil apresenta desafios no que se refere à promoção de debates problematizadores do cotidiano do trabalho, financiamento inadequado, desconhecimento da linha de base sobre a PNEPS pelos gestores e profissionais, e reprodução de práticas educativas hegemônicas do modelo tradicional de ensino como sendo EPS^(11)^. Entretanto, ao analisar as ações educativas desta revisão, fica evidente a diversidade de estratégias de ensino e sua vertente contra-hegemônica se comparada às demais ações apresentadas. Destacam-se as estratégias de ensino que contribuem para a problematização, como: discussão^(18-19,22-23)^, oficina^(20,23)^, reunião de equipe^(23)^ e análise situacional^(24)^. A abertura para o diálogo e a problematização do trabalho a partir dos saberes prévios dos profissionais são apontados como elementos diferenciais em relação ao ensino tradicional, baseado na transmissão de informações. O diálogo é um recurso que possibilita uma troca e construção contínua de significados por meio da linguagem^(50)^.

Reconhece-se a importância estratégica da EPS para fortalecer e capacitar os trabalhadores em momentos de incerteza, ao promover a aprendizagem contínua por meio da problematização da realidade, contribuindo, dessa forma, com o desenvolvimento de competências profissionais^(18-24)^. Evidencia-se sua limitação territorial e apresenta-se como um modelo potente para aplicação em outros países.

Além das ações de treinamento e EPS, foram apontadas nas publicações iniciativas de Educação Continuada (EC)^(34-35,37)^, que é entendida como uma formação após a graduação com finalidades de atualização profissional, mas que não é necessariamente realizada para atender às necessidades do cenário de trabalho como a EPS. Essa ação foi realizada para atualizar os profissionais de saúde da APS sobre a COVID-19, mas tratava-se de iniciativas formativas existentes antes da pandemia, como programas de educação para profissionais, característicos deste modelo de ação educativa. A EC não é uma ação educativa comumente utilizada no desenvolvimento de competências baseadas nas necessidades do cenário de trabalho. A EC contribuiu positivamente nos estudos em que foi citada para mudanças significativas na prática profissional no cenário da APS frente à COVID-19. As estratégias de ensino utilizadas foram: aula síncrona^(37)^, curso^(37)^, discussão^(37)^, orientação^(35)^, estudo de caso^(34)^ e caixa de bate-papo^(34)^.

É importante ressaltar que nas três tipos de ações educativas identificadas nesta revisão, foram apresentadas estratégias de ensino características de um formato educacional remoto, incluindo: aulas síncronas e assíncronas^(7,23,28-30,36-37,41,45, 46,48)^, caixa de bate-papo^(29,33-34)^, webinars^(25,28,30,33,40)^ e *webconferências* ^(22)^. A conveniência e acessibilidade das formações online foram amplamente valorizadas no contexto da COVID-19, devido ao isolamento e ao distanciamento social. Todavia, as conexões interpessoais possíveis no formato presencial são consideradas insubstituíveis^(35)^. Destaca-se que a formação remota durante a pandemia da COVID-19 possibilitou a realização de ações educativas em que o facilitador/moderador e o aprendiz podem estar em espaços geográficos distintos^(48)^, além de disponibilizar ferramentas que contribuem para o aprendizado dos profissionais de forma rápida e dialógica, como a caixa de bate-papo. A modalidade remota permite o aprendizado no ritmo individual e foi necessária durante o período de pandemia, devido ao distanciamento social^(25-35)^.

As diversas estratégias de ensino apresentadas pelos estudos têm potencialidades em desenvolver competências para atuar frente à COVID-19, uma vez que apresentam um formato horizontal e dialógico, possibilitando ao profissional um papel mais ativo em seu processo de aprendizagem. Entre essas estratégias destacam-se os estudos de caso, a discussão, a simulação, o bate-papo e o compartilhamento de experiências nas discussões e ensino prático. Aponta-se ainda a evidência do tema sobre a COVID-1 9^(7,18,20,22-24,28,31,33-36,40-41,43,45-48)^ na Figura 3 como foco na maioria das publicações, seguidos por temas relacionados, como: EPI^(20,25-26,29,32-34,39-40,43,47)^, controle de infecções^(25,27,32-33,39,42,45,47)^, organização do processo de trabalho frente à pandemia^(18,23,25)^, epidemiologia^(7,43)^, orientação sobre telessaúde^(29,33)^, higienização das mãos^(23)^ e consultoria remota^(36)^. Essa evidência era esperada devido ao contexto da revisão.

Foram identificados também, mesmo que em menor incidência, temas como: DCNTs^(35,38)^, saúde mental^(19,23)^, violência contra a mulher^(19,35)^, ética profissional^(19,41)^, alimentação^(23,38)^, saúde do idoso^(19,29)^, acolhimento humanizado^(20)^, ações de vigilância em sarampo^(20)^, avaliação neuromotora^(20)^, vigilância do desenvolvimento infantil^(21)^, técnicas de respiração^(23)^, necessidades de saúde de uma comunidade^(35)^ e introdução à APS^(48)^. Assim, é importante destacar que a APS se concentra na prevenção de doenças e agravos e na promoção da saúde, com foco na COVID-19, o que pode ter deixado lacunas no conhecimento dos profissionais sobre outras temáticas desse cenário.

As evidências não deixam claro como o desenvolvimento de competências é avaliado ou alcançado. Assim, destaca-se a necessidade de estudos primários que busquem compreender como essas competências são mobilizadas na APS. Ressalta-se que os momentos de crise e pós-crise são considerados potenciais para rupturas e avanços nas práticas sociais existentes.

### Limitações do Estudo

Este estudo apresentou limitações no que diz respeito ao número de bases de dados pesquisadas, o que pode ter contribuído para limitar o acesso a outros dados.

### Contribuições para a Área da Saúde e Enfermagem

Acreditamos que as evidências deste estudo poderão nortear profissionais e gestores de saúde para a manutenção, implementação e qualificação do cuidado ofertado aos usuários por meio de promoção de ações de aprendizagem contextualizada e problematizada pelas equipes sobre o próprio trabalho.

## CONSIDERAÇÕES FINAIS

As ações educativas realizadas para o desenvolvimento de competências profissionais mapeadas no presente estudo foram Educação Continuada (EC), EPS e treinamento. Os achados apontam para reflexões sobre a importância de desenvolver ações de aprendizagem contextualizadas no cotidiano do trabalho. Destaca-se a potencialidade da EPS para a formação e produção do trabalho em saúde, considerando seus métodos de aprendizagem que estimulam a problematização sobre os cenários de prática visando a transformação tanto do profissional quanto da própria prática.

Aponta-se também a necessidade de investimento e fortalecimento de políticas e programas, e sobretudo em estudos primários acerca de compreender as ações educativas realizadas para promover a formação contínua de profissionais para o trabalho na APS. A EPS foi considerada uma ação com potencial para a problematização e o desenvolvimento das competências profissionais para a transformação da prática no trabalho. Contudo, esse conceito é desenvolvido apenas no território brasileiro.
